# “When you use tramadol, the sperms will not come out. . .”: Unconventional strategies for avoiding unintended pregnancy among adolescents in Ghana

**DOI:** 10.1177/20503121231224660

**Published:** 2024-01-29

**Authors:** Sylvia Esther Gyan, Isaac Mensah Boafo

**Affiliations:** Department of Sociology, College of Humanities, University of Ghana, Accra, Ghana

**Keywords:** Contraceptive, unintended pregnancy, abortion, masturbation, tramadol, Ghana, adolescents

## Abstract

**Background:**

In spite of adolescents’ high knowledge about modern contraceptives, usage is low especially in sub-Saharan Africa. Little is known about what adolescents use in place of modern contraceptive methods.

**Objective:**

This paper discusses lay strategies that adolescents use in their quest to prevent unintended pregnancy.

**Methods:**

A qualitative approach was used in the study reported in this paper. The paper draws on focus group data from a larger study looking at adolescent contraceptive needs in Ghana. A purposive sampling technique was used to select 79 adolescents who participated in a focus group discussion. Eight focus group discussions were held with both male and female adolescents aged 15–19. The data were analysed using the inductive thematic analysis method after transcribing the data.

**Results:**

We found that after having sex without using condom, adolescent girls ejected sperms by using water, salt solution or ice cubes, while others engaged in pushing with pelvic floor muscles. In their bid to prevent pregnancy, for boys, engaging in masturbation and the intake of Tramadol before sex was observed. When these lay strategies fail, adolescents resorted to unsafe and illegal abortion.

**Conclusions:**

Though adolescents girls engaged in unprotected sex, they still tried to avoid pregnancy, and consequently adopted lay strategies of pregnancy prevention that do not expose them to stigma. It is therefore recommended that the Ministry of Health through the Ghana Health Service consider establishing more adolescent-friendly health centres and expanding existing ones where adolescents could easily visit to have their sexual and reproductive health needs addressed in a very confidential and non-judgemental manner.

## Background

Globally, adolescents account for 11% of all births worldwide, with about 70% of these adolescents living in low- and middle-income countries.^1^ It is estimated that 60% of all pregnancies and births among adolescent girls in low- and middle-income countries are unintended.^2^ Risky sexual behaviour among adolescents has become a global social and public health issue as it exposes them to reproductive health risks such as unintended pregnancy and sexually transmitted infections, as well as social problems such as the truncation of their education and poverty.^1^ The extant knowledge on teenage pregnancies suggest that adolescents tend to engage in unprotected sex despite the fact that most of them do not want to get pregnant.^2^ The Sustainable Development Goal 3 requires countries to ensure health and well-being for all including sexual and reproductive health (SRH) of adolescents and a commitment to end the epidemic of AIDS by the year 2030. However, adolescents continue to encounter several challenges in meeting their SRH needs, especially preventing unintended pregnancies.

In many low- and middle-income countries and the sub-Saharan African region in particular, a great lacuna exists between adolescents’ knowledge on modern contraceptives and their actual use of these contraceptives.^3–5^ Research suggests that social and cultural norms, and economic barriers limiting adolescents’ access to and use of modern contraceptives in the region and low ratings of sexual pleasure when condoms are used may explain the gap between knowledge on contraceptives and the actual use of contraceptives among this cohort of the population.^6–9^

In Ghana, knowledge of contraceptives is almost universal, with 99% of women and 99.2% of men reporting to have heard of at least one contraceptive method. In spite of this, only 19% of the sexually active population use contraceptive.^10^ The 2014 Ghana Demographic and Health Survey report shows that only 6.3% of adolescent girls aged15–19 years use modern contraceptives.^10^ The low or non-use of contraception is attributed to poor access, fear of side effects, diminished pleasure when condom is used, desire for intimacy, gender dynamics, issues of trust, misinformation and misconceptions about contraceptives.^11–13^ Adanu et al.,^11^ in a study on women in Ghana, observed that women lacked adequate information on modern contraceptives. Lack of adequate knowledge, concerns about menstrual irregularities and misinformation were also found to influence women’s use of modern contraceptive methods.^11,12^

Aside from the lack of adequate knowledge and health concerns with the use of contraceptives, one other important factor influencing the use of modern contraceptives is social norms. Social norms are the appropriate and expected behaviours of members in a society. In societies where issues of sexual experience are expected to be confidential and discreet, accessing family planning may not be common.^6^ For adolescents, accessing contraceptive is indicative of being sexually active or engaging in premarital sex, which is frowned upon in most societies in West Africa, including Ghana.^6^ These suggest that adolescents may seek for confidentiality in accessing sexual and reproductive healthcare and therefore would prefer methods that are discreet. Such preferences are influenced by the social norms in the society. Socio-cultural norms restricting communication about sexuality impede open discussion of SRH matters between adolescents and their parents. In sub-Saharan Africa, adolescent sexuality is controlled by societal norms and values that support chastity before marriage, hence parents encourage abstinence rather than the use of contraceptives.^3^ The fear of being stigmatised for accessing modern contraceptives contributes to adolescents’ low contraceptive use.^14^ To avoid stigmatisation, adolescents may come up with alternative (lay) methods for controlling their fertility that allows them to be discreet in their SRH.

Most studies across the globe as well as in Ghana tend to focus on explaining the low rates of contraceptive use among adolescents.^15–18^ For instance, Blanc and Grey^19^ suggest that factors such as observation of a period of post-partum abstinence and the issue of early pregnancy loss specifically through self-induced abortions could account for the decline in Ghana’s fertility rate. On his part, Anarfi,^20^ in his study explored the role of local herbs as a birth control method in Ghana’s fertility decline and concludes that the secrecy that comes with abortion and the fear of state laws on abortion, accounted for the little information on the use of abortion as a birth control method. Terminating pregnancies through abortion is stigmatised and sometimes even criminalised in most communities.^15,16^

The studies mentioned so far suggest that Ghanaian adolescents may be using other methods to control pregnancy and childbirth other than modern contraceptives.^15,16,19–22^ What is still unknown is whether there are other methods besides legal abortion, modern contraception and the traditional method of abstinence employed by women to manage their fertility. There seems to be limited literature on how adolescents (15–19 years) navigate risky sexual behaviours in Ghana despite their low use of modern contraception.^14,23,24^ This paper thus explores alternative strategies that adolescents (15–19 years) use in their bid to prevent unwanted pregnancies and their perceptions of these strategies.

## Methods

### Study context/area

This study was conducted in the Greater Accra Region of Ghana in August 2018. The region is reported to have the lowest teenage pregnancy rate in the country,^10^ although when categorised on the basis of residence the distribution rate is not equal across the region. Pregnancy among adolescents is highest in the rural and peri-urban areas in the Greater Accra Region. The percentage of adolescents who have started childbearing range from as low as 8% in the Greater Accra Region to as high as 18% in the Volta Region of Ghana. The Greater Accra Region is predominantly a cosmopolitan area with few rural and peri-urban areas. For this study, we focused on four peri-urban communities in two districts (Ga West District and the Ga South Districts) in the region which exhibit both urban and rural characteristics. These two districts were selected because they had some communities that were transitioning from a more rural to a more urban community. In such communities, adolescents may be grappling with traditional values and their own romantic relationships on sexual behaviour.^25^

Most households were poor and parents were either unemployed or engaged in petty trading. The level of poverty in the community made it difficult for parents to adequately meet the basic needs of their children, which, sometimes pushed girls to enter into sexual relationships with their male counterparts in exchange for financial favours. Sex and childbirth out of wedlock are frowned upon in these communities, with both parents and community members expecting adolescents to be chaste. As indicated in the study findings, the communities’ attitude towards adolescent sexual behaviour pushed girls to use discreet methods to avoid pregnancy and stigma that comes with engaging in premarital sex.

The major hospital that serves the two districts is the Amasaman District Hospital. However, within some selected communities there are community health centres that support or cater for the health needs of the people.

### Study design

This study employed a qualitative research approach using focus group discussions (FGDs) to explore strategies adolescents adopt to avoid pregnancy when they have sex without using condom. This research approach was employed to gain insights into adolescents’ use or non-use of modern contraceptives and the alternative methods they adopt to avoid pregnancy in the communities. Thus, the researchers explored the various strategies adolescents adopt as well as what they perceive others (peers) do to avoid unintended pregnancy given their low use of modern contraceptives. Adolescents who constitute the target population for this study, tend to shy away from discussions on SRH and therefore a focus group was used to solicit for responses from their own or peers’ experiences on how adolescents within their communities avoid unintended pregnancy.^26^

### Sampling and sample size

Four communities, Community Three, Community Two, Community One and Community Four in the Greater Accra Region were purposively selected because they exhibited characteristics of both rural and urban, thus making the study a unique one. The discussants were selected using the purposive sampling technique. The inclusion criteria for selecting the discussants included age (15–19 years), sex, ability to communicate in either Ga or English language, ever had sex and being a permanent resident of the community. This sampling technique was most appropriate for the study because it allowed the researchers with the help of the focal person from the Ghana Health Service to select individual adolescents who were knowledgeable about contraception, adolescent sexual behaviour and experiences in the community. The choice of sampling method and sample size was influenced by the need to gain insight into common practices and methods adolescents in these communities adopt to prevent pregnancy rather than achieving a representative sample. The sample size was thus determined by the principle of saturation. We stopped recruiting participants when additional discussion groups were yielding no new information.

### Data collection and instrument

Data were collected through FGDs with 79 adolescents (39 males and 40 females) aged 15–19 years in four communities, namely, Community Three, Community Two, Community One and Community Four in the Greater Accra Region. The selected participants for each focus group were categorised based on similar characteristics specifically age, sex, and ability to communicate in one common language.^5^ This encouraged discussants to share their common experiences and beliefs on the alternative methods they or their peers in the community adopted to prevent pregnancy before, during and after sex. Overall, eight FGDs (four groups of young men and four groups of young women) were held with 79 adolescents aged 15–19 years across the four communities, namely Community Three, Community Two, Community One and Community Four. In each community, one male and one female group discussions were held. All the groups comprised ten adolescents, except for one group with only nine young men. Seven of the FGDs were held in Ga, which is the local language of the community. The discussions in one of the focus groups with young men were held in English because it was the preferred language of the participants. The eight FGDs were moderated by the first author and assisted by a trained research assistant. With the permission of the adolescents, the discussions were audio recorded. Some of the key issues that guided the discussions bordered on factors that contribute to adolescents’ exposure to risky sexual behaviour and unintended pregnancy, adolescents’ attitudes and practices towards contraception use and adolescents’ concerns about their fertility.

All discussions were held in private places where all participants could feel comfortable, including under trees and classrooms. Each discussion lasted between 60 and 90 min. To remain focused, the discussions were steered using a guide containing the topical questions. This guide was previewed by four experts within the field of reproductive health and qualitative research prior to the data collection.

### Ethical considerations

This study was approved by the College of Humanities Ethics Review Board of the University of Ghana (ECH144/17-18). An informed consent form was designed by the researchers and participants who were aged 18–19 years were asked to sign to indicate their willingness to participate in the study. Prior to this, the purpose of the study was explained to them. For participants who were below the age of 18 years and thus considered as minors, written informed consent was first received from their legally authorised representatives, and then verbal assent was sought from the participants themselves. Legally authorized representatives and participants were also assured that the data would remain anonymous and that no aspect of the research report could be traced to any of them. All names (both participants and communities) appearing in this paper are thus pseudonyms.

### Data analysis

The data analysis process started with the transcription of all the audio recordings of discussions held in English while the recordings in Ga (local language of participants) were translated and transcribed simultaneously during the process. It is worth stating that Ga is the mother tongue of the first author, and the second author is also very proficient in the language. Together we ensured that the meanings embedded in the data did not change as we translated into Ga. The data for the study was analysed guided by the six-step model outlined by Clarke and Braun.^27^ The researchers read through all the transcripts to familiarise themselves with the content of the data. This was followed by identification of codes from the data extracts. The codes were generated by highlighting the various methods adolescents identified as alternatives for avoiding pregnancy before, during and after sex. These alternative methods were then collated into themes. After organising the codes into themes, the initial themes were further reviewed and refined by both authors. In this paper, the methods adopted by the adolescents to manage their fertility were identified and are discussed under four themes in the ensuing session. The organisation of the data and the generation of codes were done using the NVivo (version 11) software. The methods used in this paper have also been published elsewhere.^5^

## Results

### Socio-demographic characteristics of participants

The study involved almost equal numbers of male (39) and female (40) adolescents. In terms of age distribution, 74.4% of male and 80% of female discussants were minors (less than 18 years). In terms of educational attainment, only two (5.2%) of the discussants had senior high school education and one person had no formal education at all. In terms of relationship status, 9 of the males and 10 of the female discussants reported to be in a sexual relationship at the time of the study. The participants belonged to Islam (2) and Christian (77) religions. The socio-demographic characteristics of the participants have also been published elsewhere (Table 1).^5^

**Table 1.** Socio-demographic background of participants.

Socio-demographic characteristics	Young men *N* = 39 (%)	Young women *N* = 40 (%)
Age of respondents		
15	7 (18.0)	18 (45.0)
16	19 (48.7)	7 (17.5)
17	3 (7.7)	7 (17.5)
18	5 (12.8)	4 (10.0)
19	5 (12.8)	4 (10.0)
Highest level of education completed		
Senior high school	2 (5.2)	–
Junior high school	13 (33.3)	7 (17.5)
Upper primary (Class 4–6)	24 (61.5)	31 (77.5)
Lower primary (Class 1–3)	–	1 (2.5)
None	–	1 (2.5)
Relationship status		
In a relationship	10 (25.6)	9 (22.5)
Not in a relationship	29 (74.4)	31 (77.5)
Religious background		
Christian	39 (100)	38 (95.0)
Muslim	–	2 (5.0)
African traditional religions	–	–
None	–	–
Occupation		
Student	35 (89.7)	35 (87.5)
Unemployed	1 (2.5)	3 (7.5)
Employed	3 (7.7)	2 (5.0)

The study findings are discussed under four themes, ejection of sperms, masturbation, use of tramadol and abortion. The next section presents these findings.

### Ejection of sperms

Ejection of sperms from the female genitalia after unprotected sexual intercourse was one of the methods used by the female adolescents in their bid to prevent pregnancy. This was done by drinking cold water immediately after sexual intercourse believing that the sperms will mix with the water and come out as urine. Others also inserted ice cubes into their genital organ with the expectation that the cubes will melt and wash out the sperms. The efficacy of these ‘alternative’ methods of contraception have indeed, not been established scientifically and it was therefore not surprising that some indicated that these methods only worked sometimes and not all the time. What we suspect was happening was that the adolescents had low chances of getting pregnant at the times when they thought these lay strategies had worked. Below are some of the responses which reflect these post-sexual intercourse actions by the adolescents:

Some of the ladies take in very chilled water immediately after having sex and this makes them to urinate a lot. Sometimes it works other times it doesn’t so the sperms comes out through the urine (FGD Community One, female)

Yes, what I heard is that after you finish having sex, you drink a lot of water, after that you will pass a lot of urine and the sperm will come out (FGD Community Two, female).

Another lady told me that to avoid pregnancy, you can insert ice cubes into your private part for it to melt the sperms after having sex. You will pass a lot of urine, so it [sperms] will flow out as well. (FGD Community One, female)

Apart from using water or ice cubes to expel the sperms, other respondents also suggested that pushing with pelvic floor muscles after sex could also bring out the sperms after unprotected sex and thus prevent fertilisation in order to avoid pregnancy.

After sex the ladies will squat and forcefully push the sperms out from their genital organ. So even if you check you wouldn’t know that they have had sex. (FGD Community Three, female).

. . . after having sexual intercourse with the boy, whether you used a condom or not, if you know that your partner has released sperm into your vagina, you have to immediately get up and force yourself to urinate. . . and through that the sperm will all come out. Whether you feel the urge to urinate or not, you have to push hard, and, in that moment, the sperms will come out. So many of them [girls] do not use anything during the intercourse but go through this process, forcing the sperms to come out. (FGD Community Three, male)

Washing of the vagina with water or a solution of salt and water was also perceived to prevent fertilisation after an unprotected sex. Adolescent girls did this by washing the vagina after sex with water or a mixture of water and salt. This is similar to findings in Nigeria that suggest that women used salt and water to douche after having sex without a condom with the hope of avoiding getting pregnant.^28^

### Masturbation

One method that came up which most adolescents shied away from discussing was masturbation. This was revealed in the FGD with adolescent boys and girls from two communities. The discussants, though reluctant to discuss this practice explained that some adolescents satisfy themselves sexually by masturbating instead of having sex with the opposite sex to avoid pregnancy.

. . . if you do not want to impregnate a girl, I mean you put soap in your hand, you use it to . . . I mean how do you call it. . ., you use it to . . . to rub your penis [male genitalia], then you, you masturbate so that you won’t go and have sex with a girl (FGD Community Two, male).

It was also interesting to find out that apart from the well-known means of masturbation by males, adolescents employed other ‘unorthodox’ means of masturbation which included allowing a mobile phone to vibrate on one’s penis.

hmmm. . . they [peers] say. . . hmmm. . .They [boys] will put [a] phone [mobile phone] in a plastic bag and let the phone vibrate. While the phone is vibrating, they put in their penis [male genitalia] and this they said provides some level of sexual excitement. (FGD Community Two, male)

The practice of masturbation was not just limited to boys, some adolescent girls also acknowledged the practice of masturbation, albeit they claimed the practice was not common among girls in their community and female discussants disassociated themselves from masturbation, explaining that such acts were done by girls in other communities in the Greater Accra Region. However, their male counterparts suggested that some girls engaged in masturbation. The following excerpts reflect this situation:

When the boys feel for sex . . . they masturbate . . . some girls also masturbate when they feel for sex, however, it is more common in the city centre. (FGD, Community Four, female)

The girls, they satisfy themselves sexually . . . When they feel for sex, they know that they want to have sex, some of them [girls] buy candle, so that they do whatever. . . (FGD Community Two, male)

Girls’ denial of masturbation can be attributed to the norms and values of most Ghanaian societies that socialise women not to be sexually expressive.

### Use of tramadol

Probably the most remarkable finding in relation to how adolescents prevent pregnancy is the use of tramadol, which is an opiate (narcotic) analgesic. According to the adolescent boys, they sometimes swallowed tramadol pills before they had sex with girls to avoid ‘ejaculation’ during sex. This was observed in two of the study communities. In a FGD with adolescent boys from two communities, they described how they sometimes use tramadol to prevent pregnancy when they have unprotected sex with girls in the following words:

Tramadol. When you take in tramadol, the sperms will not come out, the girls will be safe from getting pregnant. (FGD Community One, male)

What I know is that if you use the medicine [tramadol] when having sex with the girl, the sperms won’t get out of your penis and enter the girl’s vagina. . . .when you take it (tramadol) and you are having sex with the girl the sperm does not get out. (FGD Community Three, male)

Although tramadol is a prescription drug in most countries, in Ghana it is common practice to access it from some vendors without a prescription, and is thus easily accessible. Additionally, the use of tramadol is not stigmatised as compared to the use of modern contraception methods. Beyond illuminating the lay strategies through which adolescents try to avoid pregnancy, this finding also highlights the fact that adolescent boys were also wary of impregnating adolescent girls they therefore devised their own strategies of avoiding pregnancy without using modern contraceptives, such as the condom. The finding is also worth noting because it also shows how adolescents inadvertently misuse certain drugs. Adolescents who frequently use tramadol have the potential to develop tolerance, dependence and addiction to the drug.^29^

### Abortion

As seen earlier, adolescents in the study intimated that these lay strategies for preventing pregnancy do not always work or have limited success. This thus makes adolescents susceptible to unintended pregnancy. It was therefore not surprising to find that abortion was one of the means of preventing childbearing. This is reflected in the following narratives:

Girls who abort pregnancy are more than those who give birth, they have traditional herbs that they use to abort it [pregnancy]. (FGD Community Three, male)

However, what is worrisome about this is the fact that these adolescents engaged in illegal and unsafe abortion thus putting their health and lives at risk. Adolescents used abortifacients sourced from pharmacy/drugstores, herbalists, friends and family members (mothers and grandmothers) and other self-acclaimed abortion specialists.

#### Use of abortion ‘specialists’

It emerged from the data that adolescent girls who got pregnant sometimes sought the services of self-styled abortion specialists in these communities. These ‘abortion specialists’ are usually persons without medical training. Their means of aborting a pregnancy ranged from using herbs and stems of plants, unprescribed pharmaceutical drugs to performing surgical procedures using crude methods in their homes and other unauthorised premises, thus putting the health and lives of these adolescent girls in danger. These are reflected in the following excerpts from participants:

Some people also do concoctions for these ladies. So, if you are pregnant and you go to these people, they will help you abort it with the concoction once you pay them. (FGD, Community Three female)

There are some people in this community whose job is to help ladies abort pregnancies. They operate in a small chamber in their homes. So, if a lady wants to abort a pregnancy, she will secretly go and see these people. (FGD Community Three, female)

#### Self-prepared abortifacients

Several abortion methods were described by the adolescents in this study. Many adolescents believed that herbs which are prepared into a mixture either for drinking or insertion into the female genitalia can be used to abort a pregnancy. These concoctions and mixtures were prepared by adolescents themselves with the intention of terminating their pregnancies.

. . . some use these herbal medicine and hard liquors, [name of the alcoholic beverage] bitters and coffee. (FGD Community One, male)

Others also resort to using the stem of plants by inserting the sharpened edge of the stick through the female genital organ to their womb to harm the growing foetus in the womb.

they [adolescent girls] use a stick known as *adaade* [jathropha plant] and they sharpen the stick to be pointed, then you dip it in a mixture and the lady would insert it in her private part [vagina] to abort the baby. It is not easy at all. And the funny thing is these ladies will get themselves pregnant again. (FGD Community Three, female)

Aside some herbs, adolescents sometimes prepared other mixtures of their own. The main ingredients used in these abortifacients are beverages with high concentration of sugar. In extreme instances, adolescents identified crude substances such as pounded glass particles that are included in the mixture with the hope of destroying the foetus in the process.

. . . malt drink mixed with mashed glass bottles, when they [pregnant girls] drink that mixture, it mashes the foetus and it comes out like blood, just like menses. Another is sugar; you take in a lot of sugar. (FGD Community Three, female)

Some [adolescents] use herbs, others use alcohol others also take in energy drinks [name of the energy drink]. Some drink [name of energy drink] others also mix Guinness with sugar, malt with sugar or fizzy drinks with sugar, coffee and [name of alcoholic beverage] to abort pregnancy. (FGD Community One, female).

High concentration of sugar was also identified by respondents as an abortifacient, which Anarfi^21^ suggests could trigger an abortion of a developing foetus.

#### Use of alcoholic beverages and pharmaceutical substances labelled as unsuitable for pregnant women

One abortifacient that was identified by all the focus group discussants, both boys and girls, was the use of alcoholic beverages/bitters as well as energy drinks. The use of alcoholic beverages as an abortifacient has become common knowledge among adolescents due to the upsurge in the number of locally manufactured alcoholic beverages as well as the numerous advertisement the producers of these beverages put up in the electronic media. It was observed that adolescents relied on the caveats in alcoholic beverages that suggested that they were not suitable for pregnant and nursing mothers. Adolescents were using these alcoholic beverages as an abortifacient because there are advertisements that suggests they were harmful to pregnant women and nursing mothers. They stated:

Nowadays from the media, they [television and radio stations] advertise some of the drinks that pregnant women are not allowed to take. Something like the [name of alcoholic beverage], pregnant women are advised not to take unprescribed drugs during pregnancy, including paracetamol, however, pregnant girls deliberately ingest such drugs to terminate pregnancy. (FGD Community Three, male)

Sometimes in the advertisements, on TV or radio, it is clearly stated that [name of alcoholic beverage] is not good for pregnant women. . . they [adolescents] will use it . . . they will take it with the hope that the pregnancy will be destroyed [aborted]. (FGD Community Two, male)

They [TV and Radio stations] have also been advertising some drugs that it’s not good for pregnant women, like Adonko bitters that if you drink it you may harm the baby. (FGD Community Two, female)

Interestingly, the study found that adolescent girls were not always the ones who ingested abortifacients in pregnant girls to abort an unintended pregnancy. Male partners had strategies of terminating an unintended pregnancy even when the adolescent girls refused to abort a pregnancy. Some adolescent girls perceived that their male partners sometimes ingest some medicines before having sex with a pregnant partner to abort an unintended pregnancy, often without the knowledge of their partners. Unfortunately, the participants were unable to provide the names of such medicines.

Some of the men, when you tell them you are pregnant, they just have lots of patience. You will be there, and he (partner) will call you to come over. He would have swallowed some tiny red pills in colour and will have sex with you again and by the time you leave and get to your house the pregnancy would have been aborted. (FGD Community One, female)

When he [partner] takes in the drugs it mixes with his sperm to cause the abortion in the lady when they have sex (FGD Community One, female)

## Discussion

This paper explored adolescents’ (15–19 years) perceptions and use of lay strategies for preventing pregnancy. Some girls adopted strategies that sought to eject sperms after having unprotected sex. This strategy is used as ‘lay emergency contraceptive’ and perceived by adolescents to have the same potency as the conventional emergency contraceptives.^28^ For the discussants, using these strategies was beneficial because it helped them to achieve their goal of avoiding unintended pregnancy which also meant avoiding stigma related to unwanted pregnancy. These are methods used in place of modern contraceptives to provide adolescent girls anonymity on their sexual behaviour. This supports available literature on why adolescents do not use modern contraceptives.^6,7^ Though adolescent girls adopt this strategy of ejecting sperms by pushing or inserting ice cubes into their genitalia immediately after having sex without condom to avoid pregnancy, the efficacy of this method has not been scientifically established. Adolescents, thus stand the risk of getting pregnant.

The practice of adolescents masturbating exists^30^ and its use as a way of avoiding pregnancy cannot be overlooked. However, in many cultures including Ghana, this nonreproductive sexual behaviour remains highly stigmatized.^31^ The silence, negative beliefs, and myths surrounding the consequences of masturbation can lead to guilt, anxiety, and shame, potentially impacting sexual health development among individuals. Despite these, masturbation continues to be practiced among unmarried adolescents (of both sexes).^31,32^ Our data suggest that this practice is fairly pervasive among the adolescents studied. We observed that the discussants were not comfortable talking about masturbation although the reactions of the other members of the discussion showed they identified with those submissions. There was a perception that both male and female adolescents turned to masturbation to abstain from having sexual intercourse with the opposite sex to avoid an unintended pregnancy.

One of the remarkable findings of this study was the fact that adolescents used tramadol as a contraceptive. The current study revealed that adolescent boys were also concerned about making a girl pregnant, hence their use of tramadol to prevent ejaculation. This finding is consistent with a recent qualitative study conducted on adolescents in South-east Nigeria where it was found that some adolescents believed that taking controlled and/or psychoactive substances before sex prevents pregnancy.^33^ Though there are no scientific bases of the methods adolescents use to avoid pregnancy, adolescents perceived using tramadol could help them control childbirth. This is an indication that adolescents have an unmet need for contraceptives^28^ and it also points to the limited knowledge that adolescents often have on how contraceptives work.^33^ Besides the unscientific bases of this method of contraception, what makes this finding worrisome is the fact that tramadol is one of the opioid drugs which is becoming widely misused among West African youth. In Ghana, though this drug is not to be sold over the counter, people are able to purchase a wide variety of prescription drugs without prescription due to weak regulatory systems and profit motives.^34^

Induced abortion is also one of the means through which women control their fertility without contraceptives.^15^ In the current study, we found that abortion was resorted to when girls end up with an unintended pregnancy. Abortions were carried out with the use of herbal mixtures which girls draank or inserted into their genitals. Similarly, Anarfi^21^ observed that women in Ghana relied on herbs as abortifacient to terminate unwanted pregnancies. He reported that some girls inserted the stalk of Jathropha plant into the womb through the vagina, to terminate pregnancies, a finding which we also reported in our study. The use of herbal medicines to induce abortion or as contraceptives have been found in other studies in Africa.^35^

The results of this study thus clearly show that adolescents put their lives and health at risk as they attempt to prevent childbirth through abortion. Indeed, induced abortion has been found to be one of the major factors directly linked to maternal deaths, accounting for between 15% and 30% of maternal deaths in the country.^7^ As Ghana seeks to achieve the SDG target of 70 per 100,000 live births it is imperative that we attend to pregnancies and pregnancy-related matters among adolescents as this category of females often try to avoid childbirth through unsafe abortion.

The use of alcoholic beverages and pharmaceutical substances labelled as unsuitable for pregnant women by adolescents as an abortifacient, is not surprising because there has been a proliferation of these drinks on popular media. Furthermore, the Ghana Food and Drugs Authority by its regulations demands that caveats such as ‘not recommended for pregnant women’ are made during advertisements of such products to promote good health. Unfortunately, adolescents are appropriating these caveats to regulate their reproductive health. More adolescents have access to this information from the media and the proliferation of such beverages and its accessibility makes adolescents subscribe to these beverages when they want to abort a pregnancy. Whereas, Biney,^15^ in her study observed that one of her respondents lost a pregnancy because she took in some alcoholic beverages unintentionally, this study observed that adolescents were taking these beverages intentionally to abort their unintended pregnancies.

## Limitations

As with the case of all qualitative research, we suggest that the findings of this study are interpreted cautiously as no generalisations could be made to the general adolescent population of Greater Accra Region. Quantitative research may be needed to establish the prevalence of the use of the lay strategies of pregnancy prevention identified by the current study. It is also possible that social desirability influenced some of the responses of respondents due to the sensitive nature of the topic.

## Implications for policy and practice

The findings of this study have implications for policy and practice. It was clear from the study that adolescents have a need for avoiding unintended pregnancies. Some of the considerations that go into the use of lay strategies for avoiding pregnancies included the need to avoid stigma. It was also clear from the findings that some adolescents are ignorant about the human reproductive system which makes them believe that certain lay strategies will be efficacious in avoiding pregnancies. We therefore suggest that:

1. There should be regular outreach programmes by community health nurses and health promoters to educate the youth in these communities on SRH matters.
2. The Ghana Health Service and the Ministry of Health must put in place adequate measures to establish adolescent-friendly health posts in communities to see to the SRH needs of adolescents in a friendly and non-judgmental manner.

## Conclusion

This study found that adolescents in Ghana adopt lay strategies (alternative methods other than modern contraceptives) to manage their SRH. These alternative methods were adopted by both male and female adolescents with the hope of avoiding unintended pregnancy. In instances where adolescent girls get pregnant, they resort to abortion methods which could generally be classified as unsafe.

Adolescents preferred methods that provide them with some level of anonymity. Ejection of sperm is a private act and the materials used are easy to acquire. Taking tramadol is also a private act since buying and taking it does not suggest any sexual activity. Again, masturbation is also a private affair. Even for self-induced abortion, adolescents adopt methods that usually does not involve a second party.

This study thus brings to the fore that adolescents have an unmet need for contraceptives. They try to avoid pregnancy despite engaging in unprotected sex and adopt methods that do not expose them to stigma. Using lay strategies to control childbirth is of importance to adolescents and therefore, relevant stakeholders should come up with programs that provide some form of confidentiality for adolescents to access reliable birth control methods. The Ministry of Health through the Ghana Health Service should consider establishing more adolescent-friendly health centres and expanding existing ones where adolescents could easily visit to have their SRH needs addressed.

## Supplemental Material

sj-docx-1-smo-10.1177_20503121231224660 – Supplemental material for “When you use tramadol, the sperms will not come out. . .”: Unconventional strategies for avoiding unintended pregnancy among adolescents in Ghana Click here for additional data file. Supplemental material, sj-docx-1-smo-10.1177_20503121231224660 for “When you use tramadol, the sperms will not come out. . .”: Unconventional strategies for avoiding unintended pregnancy among adolescents in Ghana by Sylvia Esther Gyan and Isaac Mensah Boafo in SAGE Open Medicine

sj-docx-2-smo-10.1177_20503121231224660 – Supplemental material for “When you use tramadol, the sperms will not come out. . .”: Unconventional strategies for avoiding unintended pregnancy among adolescents in Ghana Click here for additional data file. Supplemental material, sj-docx-2-smo-10.1177_20503121231224660 for “When you use tramadol, the sperms will not come out. . .”: Unconventional strategies for avoiding unintended pregnancy among adolescents in Ghana by Sylvia Esther Gyan and Isaac Mensah Boafo in SAGE Open Medicine
