# Supplementary material for: “When you use tramadol, the sperms will not come out. . .”: Unconventional strategies for avoiding unintended pregnancy among adolescents in Ghana
Source: SAGE Open Med. 2024 Jan 29;12:20503121231224660. doi: 10.1177/20503121231224660 (PMC10826370; doi:10.1177/20503121231224660)
Supplement: sj-docx-1-smo-10.1177_20503121231224660 – Supplemental material for “When you use tramadol, the sperms will not come out. . .”: Unconventional strategies for avoiding unintended pregnancy among adolescents in Ghana [file sj-docx-1-smo-10.1177_20503121231224660.docx]

COREQ (COnsolidated criteria for REporting Qualitative research) Checklist.

| **Topic** | **Item No.** | **Guide Questions/Description** | **Reported on**  **Page No.** |
| --- | --- | --- | --- |
| **Domain 1: Research team**  **and reﬂexivity** | | | |
| *Personal characteristics* | | | |
| Interviewer/facilitator | 1 | Which author/s conducted the interview or focus group? | Gyan, S.E. pg. 5 |
| Credentials | 2 | What were the researcher’s credentials? E.g. PhD, MD | PhD  This is in the author’s profile |
| Occupation | 3 | What was their occupation at the time of the study? | Lecturer  This is in the author’s profile |
| Gender | 4 | Was the researcher male or female? | Female |
| Experience and training | 5 | What experience or training did the researcher have? | Several years of training and conducting qualitative research |
| *Relationship with*  *Participants* | | | |
| Relationship established | 6 | Was a relationship established prior to study commencement? | No relationship was established prior to study commencement |
| Participant knowledge of  the interviewer | 7 | What did the participants know about the researcher? e.g. personal  goals, reasons for doing the research | Participants were informed about the profession of the researcher and the purpose of the research, prior to the research  Pg. 6 |
| Interviewer characteristics | 8 | What characteristics were reported about the inter viewer/facilitator?  e.g. Bias, assumptions, reasons and interests in the research topic | Reasons for conducting the study  Pg.6 |
| **Domain 2: Study design** | | | |
| *Theoretical framework* |  |  |  |
| Methodological orientation and Theory | 9 | What methodological orientation was stated to underpin the study? e.g. grounded theory, discourse analysis, ethnography, phenomenology,  content analysis | Phenomenology  Pg.4 |
| *Participant selection* | | | |
| Sampling | 10 | How were participants selected? e.g. purposive, convenience,  consecutive, snowball | purposive sampling  Pg. 5 |
| Method of approach | 11 | How were participants approached? e.g. face-to-face, telephone, mail,  email | Face-to-face  Pg. 6 |
| Sample size | 12 | How many participants were in the study? | 79 participants  Pg. 5 |
| Non-participation | 13 | How many people refused to participate or dropped out? Reasons? | None |
| *Setting* | | | |
| Setting of data collection | 14 | Where was the data collected? e.g. home, clinic, workplace | Private space  Pg. 6 |
| Presence of non-  participants | 15 | Was anyone else present besides the participants and researchers? | No |
| Description of sample | 16 | What are the important characteristics of the sample? e.g. demographic  data, date | Older Adolescents (15-19 yeas)  Entire paper |
| *Data collection* | | | |
| Interview guide | 17 | Were questions, prompts, guides provided by the authors? Was it pilot  tested? | Yes  Pg. 6 |
| Repeat interviews | 18 | Were repeat interviews carried out? If yes, how many? | No repeat interviews were conducted |
| Audio/visual recording | 19 | Did the research use audio or visual recording to collect the data? | Discussions were audio recorded with the consent of participants  Pg. 6 |
| Field notes | 20 | Were ﬁeld notes made during and/or after the interview or focus group? | Yes |
| Duration | 21 | What was the duration of the inter views or focus group? | 69 – 90 minutes  Pg. 6 |
| Data saturation | 22 | Was data saturation discussed? | Yes  Pg. 5 |
| Transcripts returned | 23 | Were transcripts returned to participants for comment and/or corrections | No. However, answers were repeated by the researcher for confirmation during the discussions |
| **Domain 3: analysis and**  **Findings** | | | |
| *Data analysis* | | | |
| Number of data coders | 24 | How many data coders coded the data? | Two  Pg. 7 |
| Description of the coding  tree | 25 | Did authors provide a description of the coding tree? | Yes |
| Derivation of themes | 26 | Were themes identiﬁed in advance or derived from the data? | Themes were derived from the data  Pg. 6-7 |
| Software | 27 | What software, if applicable, was used to manage the data? | Yes NVivo  Pg. 7 |
| Participant checking | 28 | Did participants provide feedback on the ﬁndings? | No |
| *Reporting* | | | |
| Quotations presented | 29 | Were participant quotations presented to illustrate the themes/ﬁndings?  Was each quotation identiﬁed? e.g. participant number | Yes  Pg. 8-13 |
| Data and ﬁndings consistent | 30 | Was there consistency between the data presented and the ﬁndings? | Yes  Pg. 8-13 |
| Clarity of major themes | 31 | Were major themes clearly presented in the ﬁndings? | Yes  Pg. 8-13 |
| Clarity of minor themes | 32 | Is there a description of diverse cases or discussion of minor themes? | Yes  Pg. 8-13 |
