# Supplementary material for: “When you use tramadol, the sperms will not come out. . .”: Unconventional strategies for avoiding unintended pregnancy among adolescents in Ghana
Source: SAGE Open Med. 2024 Jan 29;12:20503121231224660. doi: 10.1177/20503121231224660 (PMC10826370; doi:10.1177/20503121231224660)
Supplement: sj-docx-2-smo-10.1177_20503121231224660 – Supplemental material for “When you use tramadol, the sperms will not come out. . .”: Unconventional strategies for avoiding unintended pregnancy among adolescents in Ghana [file sj-docx-2-smo-10.1177_20503121231224660.docx]

**FOCUS GROUP DISCUSSION GUIDE**

1. Socio-demographic background of discussants
2. Knowledge on contraceptives
3. When contraceptives are used?
4. Why contraceptives are used?
5. Which contraceptive methods do adolescents in the community know/use?
6. Identify and share knowledge on the various traditional contraceptive methods
7. Why do adolescents use the traditional methods?
8. Identify and share knowledge on the various modern contraceptives methods you know
9. Which modern contraceptive method is mostly used by adolescents in the community?
10. Why do adolescents use the modern methods?
11. How do adolescents get to know about the various modern contraceptive methods?
12. How do adolescents get to know about the various modern contraceptive methods?
13. How accessible are the modern contraceptives?
    1. Places (hospital, clinic, CHPS compounds, Chemical shops, Pharmacy etc.
    2. People (Boyfriend, girlfriend, peers, parents etc.)
    3. Any other source
14. Which contraceptive methods are preferred?
15. Reasons why a contraceptive method is preferred over the other:
    1. Cost
    2. Knowledge
    3. Accessibility
    4. Social context e.g. allows for secrecy
16. When do you use any contraceptive method and why?
17. Who initiates the use of a contraceptive methods and why?
18. How often do you use contraceptives?
19. Do you always plan when you are going to have sex?
20. How do you plan?
21. What is the community’s position on sex out of wedlock for adolescents? (Parents, neighbours and peers)
22. How will the community respond to your sexual behaviour? (Parents, neighbours and peers)
23. Does your community’s reaction to your sexual behaviour affects the strategies you use to manage your fertility?
24. What do you do after you have had unprotected sex?
25. What do you do when you find out that you are pregnant after having sex?
26. Can you tell when you have STIs?
27. How you do you know you have an STI?
28. What do you when you find out you have STI?
29. Discuss your experiences with any of the contraceptive method
